# GlideScope and Frova Introducer for Difficult Airway Management

**DOI:** 10.1155/2013/717928

**Published:** 2013-08-07

**Authors:** Alessandra Ciccozzi, Chiara Angeletti, Cristiana Guetti, Roberta Papola, Paolo Matteo Angeletti, Antonella Paladini, Giustino Varrassi, Franco Marinangeli

**Affiliations:** ^1^Anesthesiology and Pain Medicine, Department of Life, Health and Environmental Sciences, University of L'Aquila, Viale San Salvatore, Edificio 6, 67100 L'Aquila, Italy; ^2^Operative Unit of Anesthesiology, Intensive Care and Pain Medicine, Civil Hospital “G. Mazzini” of Teramo, Piazza Italia, 64100 Teramo, Italy

## Abstract

The introduction into clinical practice of new tools for intubation as videolaringoscopia has dramatically improved the success rate of intubation and the work of anesthesiologists in what is considered the most delicate maneuver. Nevertheless intubation difficulties may also be encountered with good anatomical visualization of glottic structures in videolaringoscopia. To overcome the obstacles that may occur both in a difficult provided intubation such as those unexpected, associated endotracheal introducer able to facilitate the passage of the endotracheal tube through the vocal cords into the trachea may be useful. We report 4 cases of difficult intubation planned and unplanned and completed successfully using the GlideScope videolaryngoscope associated with endotracheal Frova introducer.

## 1. Introduction

Difficult airway management is a major task for anesthesiologists [1, 2]. Failure in airway management indeed, is a major cause of mortality and morbidity in the setting of anesthesiology and intensive care units [3, 4].

The GlideScope (GS) is a videolaryngoscope (VLS), the last generation of intubation devices available in clinical practice in the last decade. GS provides an indirect airway view, improves the assessment of Cormack-Lehane score, and does not require a specific training [5, 6].

 Recent studies underline the advantages of VLS in the management of predicted difficult airway [7, 8] as well as prehospital emergencies [9].

Unfortunately, the direct laryngeal view provided by VLS does not always assure the correct insertion of endotracheal tube (ETT), due to the 60-degree angle in the distal portion of GS blade, that tends to hamper the passage of the ETT from oropharynx to trachea. To facilitate the placement of the ETT, a rigid stylet shaped with the same angle as the blade, the GlideRite stylet (GRs), has been made up. Recently, the most suitable characteristics of the introducer have been largely debated: gum elastic bougie, rigid stylet, malleable stylet, and several experiences have been published with different endotracheal introducer utilized in combination with VLS to facilitate intubation maneuver [10–14].

We report our clinical experience in 4 patients, three characterized by potential and one by unexpected difficult intubation, in whom videolaryngo-GlideScope (VLGS) combined with Frova bougie has been used to facilitate endotracheal intubation.

## 2. Case  1

**Figure 1:**
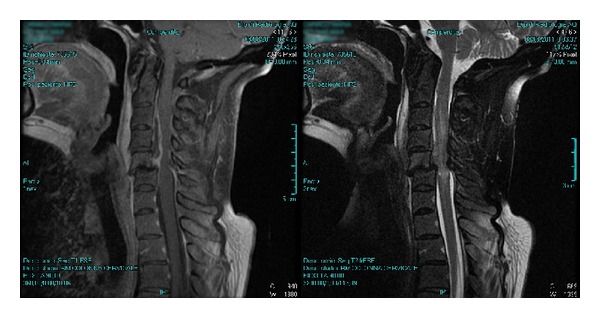
MRI Case  1: altered signal intensity with synovial effusion in interapophyseal joints C5-C6 and C4-C5, associated with extensive alteration of signal paravertebral soft tissues on the left paravertebral front and retropharyngeal from C1 to C7; there is also extensive alteration signal in correspondence with the epidural space anterior and lateral left from C3 to C6 and more evident in C4-C5 left side. Segmental stenosis of the spinal canal at C5-C6, with compression on the spinal cord and the abolition of the subarachnoid space perimedullary, is evident. The spinal cord has altered signal intensity level from C3 to C6. Framework compatible with the involvement of nature-infectious inflammatory type septic arthritis interapophyseal with paravertebral and epidural abscesses and myelopathy.

A 61-year-old woman (BMI: 22.6 kg/m^2^) was urgently admitted to the anesthesiological evaluation before undergoing the intervention of spinal decompression of cervical C3–C6 stenosis secondary to a paravertebral *Staphylococcus aureus* abscess (Figure 1). The patient, in ASA physical status III, was affected by rheumatoid arthritis (RA) and treated with biologic immunomodulators and monoclonal anti-TNF antibodies. Evaluation of morphofunctional indices predictive of difficult intubation and mask ventilation showed a degree of potential difficulty: complete normal teething, interincisor gap >3 cm, extension of the atlooccipital joint <30°, thyromental distance >6.5 cm, sternomental distance <12.5 cm, upper lip bite test = 1 (ULTB), Mallampati score = 3, and neck circumference <40 cm. No additional indices of difficult intubation and ventilation were present, such as history of snoring, obstructive sleep apnea syndrome (OSAS), dental or mandibular abnormalities, and macroglossia. Premedication with midazolam 2.5 mg and atrophine 0.5 mg was administered. General anesthesia was induced by intravenous administration of propofol 2 mg/kg, rocuronium bromide 1.2 mg/Kg, fentanyl 0.05 mg, and subsequent infusion of remifentanil at incremental doses; denitrogenation was previously obtained by the administration of pure oxygen in mask for 3 min. 

An adequate muscle relaxation was obtained; considering the severe neck stiffness and impaired movement of cervical structures, worsened by the abscessual lesion and by the risk of bone fracture due to preexisting arthrodystrophic disease, we straightly proceeded to indirect laryngoscopy with GS. The procedure, performed by an expert anesthesiologist, revealed a Cormack-Lehane score of IIb, that was unmodified by the back upward right-sided pressure maneuver (BURP) on the thyroid-cricoid cartilages, as well as a marked decrease of the oral and hypopharyngeal space. Indirect laryngoscopy did not allow to correctly place the ETT preloaded with the manufacturer's preconfigured stylet GRs even after the BURP maneuver. A second, successful attempt was made by using the Frova introducer after the BURP maneuver. The times recorded for each attempts are summarized in Table 1. The values of hemodynamic parameters (systolic and diastolic blood pressure, SBP and DBP, and heart rate, HR) and arterial O_2_ saturation were summarized in Table 2. The second attempt was performed after the patient had been reoxygenated and remifentanil dose had been gradually increased up to 0.20 mcg/kg/min in order to minimize the hemodynamic effects of repeated intubation maneuvers. After neurosurgical procedure, which overall lasted for 170 min, the patient was extubated without any complication; no lesions of the pharyngolaryngeal structures were observed. The patient complained of mild pharyngodynia when she woke up in the recovery room.

## 3. Case  2

**Figure 2:**
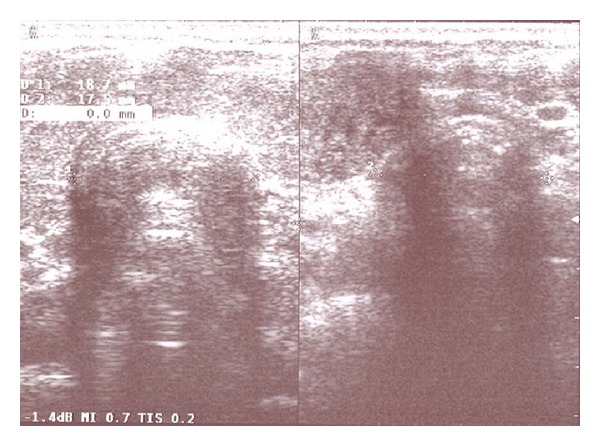
Neck and thyroid ultrasound examination (Case  2): trachea with a reduction of the transverse diameter (17.5 mm) in the distal cervical tract and dislocation to the left of the trachea.

A 48-year-old woman (BMI: 19.92 kg/m^2^) presented to the anesthesiological evaluation before undergoing total hysterectomy and salpingectomy due to uterus fibromatosis. The patient, in ASA physical status I, was affected by nodular thyreopathy in stable clinical phase; she denied any anesthaesiological complication during previous surgical interventions. The evaluation of morphofunctional indices predictive for difficult intubation and mask ventilation revealed an apparently normal grade of difficulty: normal and complete teething, interincisor gap >3 cm, good neck mobility, thyromental distance >6 cm, sternomental distance >12, ULTB = 1, Mallampati score = 2, and neck circumference <40 cm. No additional indices predictive of difficult intubation were present, in particular history of snoring, OSAS, dental or mandibular abnormalities, and macroglossia. The ultrasonographic examination of the neck revealed that trachea was narrowed in its cervical tract (transverse diameter = 17.5 mm) and displaced to the left (Figure 2). The patient was premedicated with midazolam 5 mg and atrophine 0.5 mg. Denitrogenation was obtained by the administration of pure oxygen in mask for 3 min; general anesthesia was induced by intravenous administration of propofol 2 mg/kg, rocuronium bromide 0.6 mg/kg mg, and remifentanil 0.05–0.25 micg/kg/min. After an adequate muscle relaxation had been obtained, a resident anesthesiologist proceeded to the first direct laryngoscopy, that failed due to the presence of a Cormack-Lehane score IV, that was unmodified by the BURP maneuver. A second direct laryngoscopy attempted by the senior anesthesiologist again failed, due to the persistence of a Cormack-Lehane score IV, unmodified by the BURP maneuver. Then, a third intubation was attempted with the GlideScope: indirect laryngoscopy revealed a Cormack-Lehane score IIb; however, endotracheal tube preloaded with the manufacturer's preconfigured GRs could not be correctly placed, even after the BURP maneuver. Finally, a fourth procedure was successfully performed after substituting the GRs with the Frova introducer. The times recorded for each attempts are summarized in Table 1. The values of SBP, DBP, HR, and arterial O_2_ saturation were reported in Table 2. The second, third, and fourth procedures were attempted after the patient had been reoxygenated and remifentanil dose gradually increased up to 0.25 mcg/kg/min in order to minimize the hemodynamic effects of repeated maneuvers. Overall, the gynecological intervention lasted for 90 minutes; subsequently, the patient was administered sugammadex 2 mg/kg and easily extubated; no pharingolaryngeal lesions were observed at the inspection of the oral cavity. The patient complained of mild pharyngodynia when she woke up in the recovery room.

## 4. Case  3

A 61-year-old woman (BMI: 17.6 g/m^2^) arrived to the anesthesiological evaluation before undergoing the intervention of microdiscectomy due to L_5_-S_1_ disc herniation. The patient, in ASA physical status I, did not refer to relevant diseases in her medical history, with the exception of an intervention of cervical arthrodesis with a normal perioperative follow-up. The evaluation of morphofunctional indices predictive for difficult intubation and mask ventilation indicated a degree of potential difficulty: complete and stable teething, interincisor gap >3 cm, reduced flexion-extension properties of cervical spine, thyromental distance >6.5 cm, sternomental distance <12.5, ULTB = 2, Mallampati score = 3, and neck circumference <40 cm. No additional indices of difficult intubation and mask ventilation were present, including history of snoring, obstructive sleep apnea syndrome, dental and mandibular abnormalities, and macroglossia. Premedication with midazolam 2.5 mg and atrophine 0.5 mg was administered; general anesthesia was induced by intravenous administration of propofol 2 mg/kg, rocuronium bromide 0.6 mg/kg mg, and remifentanil 0.05–0.25 mcg/kg/min,after denitrogenation had been obtained by the administration of pure oxygen in mask for 3 min. After an adequate muscle relaxation had been obtained, a junior anesthesiologist proceeded to the first direct laryngoscopy, in the presence of a Cormack-Lehane score IV, that was unmodified after the BURP maneuver. A second intubation was attempted with GS device; although indirect laryngoscopy performed by the resident anesthesiologist revealed a decrease of Cormack-Lehane score from III to IIb after BURP maneuver, the endotracheal tube preloaded with the manufacturer's preconfigured GRs could not be correctly placed. Thus, the senior anesthesiologist proceeded to the third successful attempt after the BURP maneuver, by replacing preconfigured GRs with the Frova introducer. The times recorded for each attempts are summarized in Table 1. The values of SBP, DBP, HR, and arterial O_2_ saturation were reported in Table 2. The second and third attempts were performed after the patient had been reoxygenated and remifentanil dose gradually increased up to 0.25 mcg/kg/min, in order to minimize the hemodynamic effects induced by repeated intubation maneuvers. At the end of the neurosurgical procedure that globally lasted for 185 min, the patient was easily extubated; no lesions of pharingolaryngeal structures were found at the inspection of the oral cavity. The patient complained of mild pharyngodynia when she woke up in the recovery room.

## 5. Case 4

A 62-yr-old woman (BMI: 39 kg/m^2^) presented to anesthesiological evaluation before undergoing the intervention of spinal decompression due to dorsal stenosis. The patient, in ASA physical status II, was affected by metabolic syndrome, hypertension, obesity, and dyslipidemia. The evaluation of morphofunctional indices predictive of difficult intubation and mask ventilation indicated a degree of potential difficulty: incomplete teething and mobile upper teeth, interincisor gap >3 cm, slightly reduced flexion-extension properties of cervical spine, thyromental distance >6.5 cm, sternomental distance <12.5 cm, sterno-thyroid distance = 6 cm, ULTB = 2, Mallampati score = 3, and neck circumference of 47 cm. No additional indices predictive of difficult intubation and mask ventilation were present, in particular history of snoring, OSAS, dental or mandibular abnormalities, and macroglossia. A premedication with midazolam 5 mg and atrophine 0.5 mg was administered; general anesthesia was induced by intravenous administration of propofol 2 mg/kg, rocuronium bromide 0.9 mg/kg mg, and remifentanil 0.05–0.25 mcg/kg/min, after denitrogenation had been obtained by the administration of pure oxygen in mask for 3 min. After an adequate muscle relaxation had been obtained, an expert anesthesiologist proceeded to the first direct laryngoscopy and evidenced a Cormack-Lehane score IV, that was unmodified after the BURP maneuver. Due to the presence of severe neck stiffness and the tendency of the patient to rapidly desaturate, a second intubation maneuver was attempted with GS device. Although indirect laryngoscopy revealed a Cormack-Lehane score II after BURP maneuver, endotracheal tube preloaded with the manufacturer's preconfigured GRs could not be correctly placed. Finally, after the BURP maneuver a third attempt was successfully carried out, after preconfigured GRs were replaced with Frova introducer. The times recorded for each attempts are summarized in Table 1. The hemodynamic and saturation in oxygen were summarized in Table 2. The second and third maneuvers were performed after the patient had been reoxygenated and remifentanil dose gradually increased up to 0.25 mcg/kg/min, in order to minimize the hemodynamic effects of repeated intubations. At the end of the neurosurgical procedure, which globally lasted for 240 min, the patient was extubated without any complication. The patient did not complain of pharyngodynia when she woke up in the recovery room.

## 6. Discussion

A recent meta-analysis of 17 studies including 1998 patients has confirmed the advantage of GlideScope over direct laryngoscopy for a better glottic visualization in patients with potential or simulated difficult airways [15].

Despite the excellent visualization of the glottis and vocal cords with GS-VLS the anesthesiologist must be aware of the possible difficulties in the introduction of the ETT through these anatomical structures. The main difficulty intubation is the need for extreme bending of the distal portion of the ETT. The bevel of the distal portion of the ETT can hang on the arytenoid cartilages or impact against the front wall of the larynx, preventing and making it difficult to guide the ETT through the vocal cords into the trachea. This is particularly difficult in patients with unanticipated anterior airway, protruding upper teeth, small chin, and thyroid-mental distance less than 6 cm, where the angle acute ETT can prevent the progress and the insertion of the ETT through the vocal cords [16, 17].

It has been suggested that the use of a flexible stylet which allows the regulation of the ETT can be useful to decrease time to intubation and increase the success rate [18, 19].

The clinical cases previously reported prove that GlideScope combined with Frova introducer allowed a successful intubation of patients with potential or unexpected difficult airway. Frova introducer, indeed, enhanced endotracheal intubation and GS allowed the correct placement of the introducer tip just before vocal cords, thus minimizing the risk of endobronchial injury, as previously reported by Heitz and Mastrando [20]. Frova introducer is the gum elastic boogie routinely used in our hospital; in combination with GS, it may overcome some VLS limitations and offer multiple advantages, in accordance with Sharma; its distal tip anteriorly angulated at 30° enhances the endotracheal insertion of the device; the device is easily handled and atraumatic [21]. Moreover, the device is provided with an adaptor that allows to oxygenate the patient or to change the endotracheal tube in case of inadequate caliber. Finally, Frova introducer may overcome a common limitation of available videolaryngoscopes, namely, the limited room available for the manipulation and insertion of endotracheal tube [22].

The combination of both devices allowed us to succeed in difficult cases, such as the patient with rheumatoid arthritis and cervical abscess. In this case, indeed, the stiffness of the cervical spine associated with edema was causing a significant reduction of retropharyngeal space. The success of indirect laryngoscopy in this case confirmed previous reports by Malik et al. about the GlideScope use in patients with immobilized cervical spine [23]. Indeed, GS, like other VLS, does not require an alignment of the oral, pharyngeal, and laryngeal axes to see the glottis. Has been demonstrated that patients with ankylosing spondylitis (AS) can be successfully intubated by GS [24, 25]. This latter may be a better alternative in most cases, compared with the Macintosh laryngoscope, for intubating patients with restricted movement of the head and neck, either by pathological changes or by active stabilization, while the fibreoptic intubation may be often difficult in these patients [26]. Furthermore, the GS offers some advantages compared to the fiberscope: it can also be used after having induced general anesthesia, as in the present cases; it does not require a long learning curve for the expert anesthesiologist; it can be very useful in the patients who refuses awake intubation. A further aspect could be stressed, the availability, in our hospital, of rocuronium bromide and sugammadex. Both have allowed a safer management of potentially difficult airways, in particular in the patient with RA, operated in urgency. Indeed, the administration of rocuronium bromide (1.2 mg/kg) is able to ensure more rapid onset of a profound neuromuscular block compared to succinylcholine, while the sugammadex (16 mg/kg) allows a rapid recovery from a deep neuromuscular blockade [27]. Therefore, sugammadex should always be available, as escape drug, when using rocuronium bromide.

The combination of VLS with Frova introducer offers major advantages in predicted difficult intubations, as underlined by Añez Simón et al. [28] and Asai [29] At difference from studies by Añez Simón et al. and Asai, who used the McGrath VLS and Pentax-AWS, respectively, in our present series we used GS, the only VLS available in our hospital.

In Asai's report, awake nasal intubation was performed in the presence of *“unstable”* neck and reduced retropharyngeal space. In particular, in one of the three patients with cervical spine injury, a posterior laryngeal wall hematoma at C4–C7 level was reducing supraglottic space and causing a reactive larynx oedema: endotracheal tube was correctly placed in this patient after the tube itself was inserted on the tracheal introducer by the nasal route and the glottis was visualized by the orally inserted Pentax AWS. The author, therefore, recommends that tracheal introducer should be used also for nasal intubation, particularly when the tip of the nasal tube is deviated from the glottic target [29].

In our experience, in three patients with potential difficult intubation the maneuver was performed after induction of general anesthesia and a careful evaluation of predictive indices of difficult intubation and mask ventilation, in accordance with the literature recommendations [30, 31].

After preoperative evaluation of difficult intubation and mask ventilation indices, an expert anesthesiologist could optimize airways management by the sequential use of both GlideScope and Frova introducer, on the basis of the Cormack-Lehane score derived from the direct and indirect laryngoscopy.

Frova introducer was used according to Italian guidelines for difficult airway management, which recommend an early use of this device in case of difficult laryngoscopy [32].

Undoubtedly, the expertise of both devices was fundamental for the success of the intubation procedure, as the operator optimized the synergistic use of both instruments, in particular in the presence of unexpected difficult intubation, which represents the most serious challenge for the anesthesiologist.

Additional considerations derive from the analysis of the hemodynamic parameters: our anesthesiological schedule could adequately control the values of BP and HR after repeated intubation attempts; the rate-pressure product (RPP), indeed, never exceeded the 22000 threshold value for myocardial ischemia [33].

The control of hemodynamic responses is fundamental during laryngoscopic and intubation procedure with VLS and GS, particularly when these highly stressful maneuvers have to be repeated. It should be pointed out that the duration of the procedure is longer with GS, which, in this respect, does not offer major advantages over the Macintosh laryngoscope [34].

This effect may be related to several factors, including the wider GS blade (18 mm thickened and posteriorly squared) that strongly stimulates posterior tongue and pharyngeal structures; due to its larger dimensions, GS blade requires that an additional upward lifting force is exerted so as to create enough room for the passage of the tracheal tube. Moreover, the stylet, aimed at maintaining the desired curvature of endotracheal tube, tends to prolong the intubation time and to more extensively stimulate laryngotracheal structures [34].

In addition, using the technique described in our experience, you can follow directly under the display GlideScope-assisted introduction of the endotracheal tube through the vocal cords, minimizing the trauma of the airway.

## 7. Conclusions

The GlideScope videolaryngoscope combined with Frova introducer allowed us to successfully manage predicted as well as unpredicted difficult intubations. Specific knowledge of advantages and disadvantages of each device, expertise in their use in clinical practice, and preoperative evaluation of predictive indices of difficult intubation were fundamental for the successful procedure. The gum elastic bougie used with the videolaryngoscope is therefore a complementary association of old and new technologies, which can be combined to rapidly achieve ETT in cases of predicted and unexpected difficulty.

GS and other commercially available VLS do not require a specific training: this aspect may profoundly impact airways management, either in the setting of intra- or extra-hospital difficult airway intubation as in the educational field.

## Figures and Tables

**Table 1:** Time of endotracheal intubation attempts performed with different devices used in our experience.

Case	Direct laryngoscopy performed by resident anesthesiologist		Direct laryngoscopy performed by expert anesthesiologist		GlideScope and GRs		GlideScope and Frova introducer	
	Time (seconds)	EI	Time (seconds)	EI	Time (seconds)	EI	Time (seconds)	EI
1					40	Failed	25	Yes
2	160	Failed	110	Failed	75	Failed	45	Yes
3	30	Failed			47	Failed	45	Yes
4	25	Failed			50	Failed	35	Yes

GRs: GlideRite stylet; EI: endotracheal intubation.

**Table 2:** Hemodynamic parameters and saturation in oxygen during endotracheal intubation attempts.

Case	Before anaesthesia induction				First attempt				Second attempt				Third attempt				Fourth attempt			
	SBP	DBP	HR	SaO_2_	SBP	DBP	HR	SaO_2_	SBP	DBP	HR	SaO_2_	SBP	DBP	HR	SaO_2_	SBP	DBP	HR	SaO_2_
1	130	70	60	98%	147	82	84	93	100	52	94	97%								
2	120	70	78	100%	136	75	84	95%	142	77	86	93%	148	82	88	98%	145	80	86	97%
3	120	70	70	100%	124	80	74	98%	146	91	81	100%	137	61	100	98%				
4	124	78	70	96%	114	58	92	94%	96	44	92	98%	90	51	68	98%				

SBP: systolic blood pressure (mmHg); DBP: diastolic blood pressure (mmHg); HR: heart rate (bpm); SaO_2_: saturation in oxygen (%).

## Abbreviation

AS: Ankylosing spondylitis
BMI: Body mass index
BP: Blood pressure
BURP: Back upward right-sided pressure maneuver
DBP: Diastolic blood pressure
EI: Endotracheal intubation
ETT: Endotracheal tube
GS: GlideScope
GRs: GlideRite stylet
HR: Heart rate
OSAS: Obstructive sleep apnea syndrome
RA: Rheumatoid arthritis
RPP: Rate-pressure product
SBP: Systolic blood pressure
ULTB: Upper lip bite test
VLGS: Videolaryngo-GlideScope
VLS: Videolaryngoscope.
